# La fracture de verge: à propos de six observations au CHU Sanou Souro de Bobo-Dioulasso, Burkina Faso

**DOI:** 10.11604/pamj.2019.33.257.19452

**Published:** 2019-07-26

**Authors:** Abdoul-Karim Paré, Adama Ouattara, Gnimdou Botcho, Brahima Kirakoya, Fasnewendé Aristide Kaboré, Amidou Bako, Delphine Yé, Dramane Bayané, Mireille Konaté, Timothée Kambou

**Affiliations:** 1 Service d'Urologie Centre Hospitalier Universitaire Sanon Souro, Bobo-Dioulasso, Burkina Faso; 2 Service d'Urologie Centre Hospitalier Universitaire Yalgado Ouédraogo, Ouagadougou, Burkina Faso

**Keywords:** Fracture de verge, corps caverneux, rupture de l´urètre, Penile fracture, corpus cavernosum, urethral rupture

## Abstract

La fracture du pénis est une urgence urologique rare, définie comme une rupture traumatique de la tunique albuginée du corps caverneux. Elle touche essentiellement le sujet jeune au cours de l'activité sexuelle. Le but de ce travail était de rapporter les résultats de la prise en charge de 06 cas de fracture de verge observés dans le service d'Urologie-Andrologie du Centre Hospitalier Universitaire Sanon Souro (CHUSS) de Bobo-Dioulasso. Il s'agissait de six patients d'âge moyen de 38,3 ans, reçu pour tuméfaction douloureuse de la verge pour 04 cas et d'urétrorragie persistante pour 02 cas au décours de faux pas du coït ou de manipulation forcée de la verge. La tuméfaction douloureuse de la verge avec la verge en “aspect d'aubergine” a été le principal signe retrouvé. Le traitement a consisté en une évacuation de l'hématome intra-caverneux suivi d'une albuginorraphie pour 05 cas et pour un cas d'un traitement conservateur. Les suites opératoires ont été simples pour l'ensemble des patients.

## Abstract (en)

Penile fracture is a rare urologic emergency, defined as a traumatic rupture of the tunica albuginea of the corpus cavernosum. It mainly affects the young subjects during sexual intercourse. The purpose of this study was to report treatment outcomes in 6 patients with penile fracture observed in the Department of Urology-Andrology, Souro Sanou University Hospital of Bobo-Dioulasso. The study involved six patients with an average age of 38.3 years admitted in the hospital with painful penile swelling (4 cases) and persistent urethrorragia (2 cases) after wrong coital movement or forced manipulation of the penis. Painful swelling of the penis with penis simulating the appearance of an aubergine was the main sign found. Treatment was based on evacuation of the intracavernous haematoma followed by albuginorraphy in 5 cases and conservative treatment in 1 case. All patients had an uneventful postoperative course.

## Introduction

La fracture du pénis est une urgence urologique rare, définie comme rupture traumatique de la tunique albuginée du corps caverneux. Cette affection cible essentiellement les adultes jeunes pendant l'activité sexuelle [1, 2]. Elle est observée sur le pénis en érection. Les lésions concernent les corps caverneux essentiellement mais une association à une rupture de l'urètre est parfois retrouvée [3]. La rupture isolée du corps spongieux est une entité très rare et est également souvent accompagnée d'une rupture de l'urètre [4]. Le but de ce travail est de rapporter les résultats de la prise en charge de 06 cas de fracture de verge observés dans le service d'Urologie Andrologie du Centre Hospitalier Universitaire Sanon Souro (CHUSS) de Bobo-Dioulasso.

## Méthodes

Il s'est agi d'une étude rétrospective portant sur tous les cas de fracture de verge recensés en 05 ans 02 mois de janvier 2014 à février 2019 au CHUSS de Bobo-Dioulasso. Les paramètres étudiés étaient l'âge, les circonstances de survenue, le motif de consultation, le mécanisme, les données de l'examen physique, le délai de la prise en charge, le traitement institué, les suites opératoires et la fonction sexuelle après traitement. Pour toutes les variables numériques, une moyenne a été calculée et les extrêmes définies.

## Résultats

**Figure 1:**
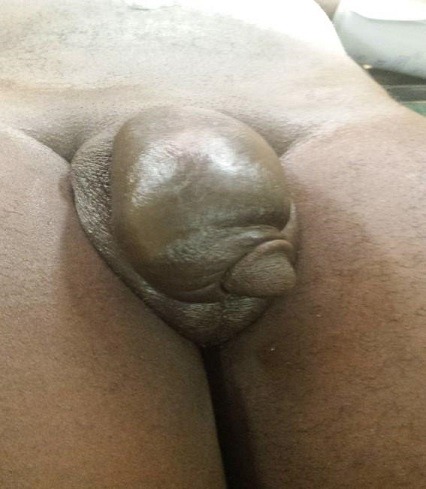
Verge œdématiée d’aspect aubergine

**Figure 2:**
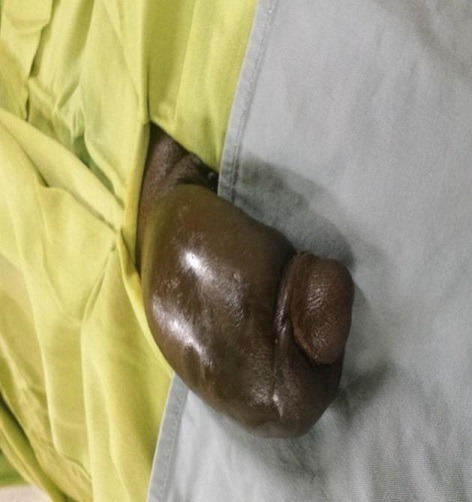
Verge tuméfiée

Au total, six (06) cas de fracture de verge ont été recensés au cours de notre période d'étude. L'âge moyen des patients était de 38,3 ans avec des extrêmes de 30 ans et 43 ans. Le délai moyen de prise en charge était de 46,3 heures soit environ 02 jours avec des extrêmes de 03 heures et de 408 heures. Le motif de consultation était la tuméfaction douloureuse de la verge dans 04 cas, la tuméfaction de la verge associée à une urétrorragie dans 01 cas et l'urétrorragie isolée dans 01 cas. Quant à la circonstance de survenue, il s'agissait du faux pas du coït dans 04 cas et une manipulation forcée sur la verge dans 02 cas. L'examen physique a retrouvé une verge tuméfiée avec déviation donnant « l'aspect d'aubergine » dans 04 cas comme attesté par les Figure 1 et Figure 2. De plus, l'examen a permis de noter une verge d'aspect normal associée à une urétrorragie dans 01 cas et une verge tuméfiée associée à une minime urétrorragie dans 01 cas.

**Figure 3:**
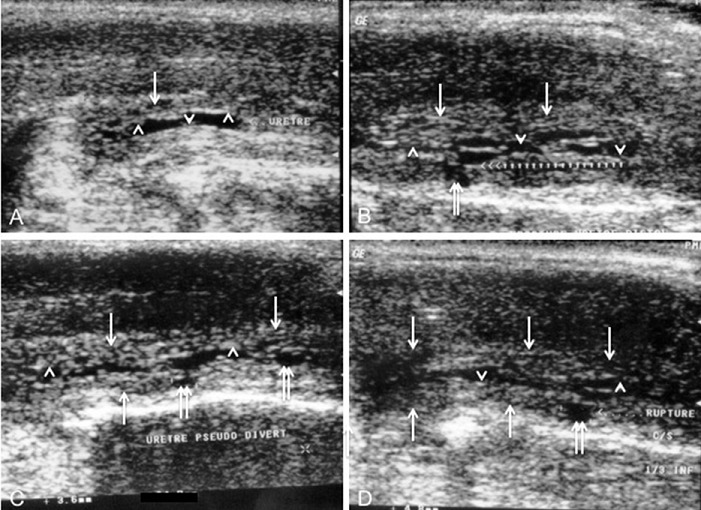
L´échographie montre une irrégularité étendue, anfractueux des parois de l´urètre avec dédoublement pariétal par endroits (A, B, C, D, tête de flèche), un hématome circonférentiel (A, B, C, D, flèche) et deux diverticules profonds du 1/3 distal (B, C, D, double flèches) témoignant d´une fracture du corps spongieux associée sans atteinte des corps caverneux

**Figure 4:**
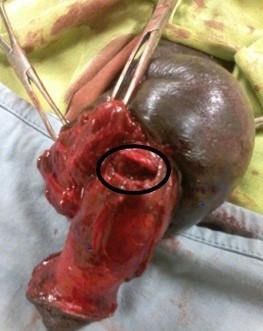
Identification de la rupture du corps caverneux (CC)

**Figure 5:**
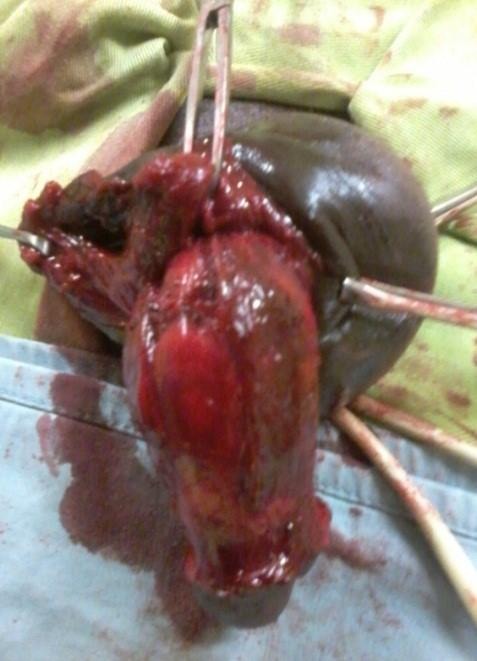
Albuginorraphie

Le diagnostic a été posé après l'examen clinique dans 05 cas. Dans un cas, une échographie de la verge puis une urétrocystographie rétrograde (UCR) ont été réalisées; ce qui a permis de conclure à une rupture partielle de l'urètre associée à une contusion du corps spongieux (Figure 3). Le traitement a constitué après une rachi anesthésie en une incision circonférentielle balano-préputiale suivi d'une dénudation complète de la verge avec accès au corps caverneux et un débridement des tissus des corps caverneux (CC) avec évacuation de l'hématome intra caverneux. L'exploration a permis l'identification du défect matérialisé par la Figure 4 suivi de l'albuginorraphie dans 05 cas (Figure 5).

**Figure 6:**
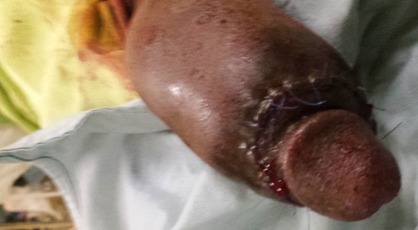
Suture de l´incision coronale

**Figure 7:**
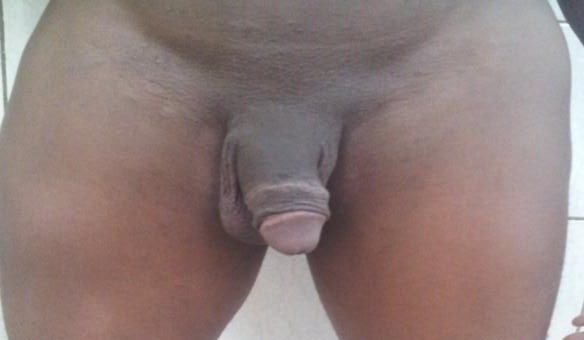
Aspect verge après 6 mois

Un traitement conservateur par la mise en place d'une sonde urétro-vésicale dans un but d'hémostase, de maintien de la lumière urétrale associé à une antibioprophylaxie a été fait dans un cas. Les suites opératoires ont été simples dans les 05 cas opérés avec une verge d'aspect normal à 01 mois post opératoire avec une absence de symptomatologie urinaire (Figure 6). À l'ablation de la sonde chez le patient ayant bénéficié d'un traitement conservateur, l'évolution était favorable (Figure 7). Une reprise des activités sexuelles a été notée au bout de 03 mois dans les 06 cas. Le Tableau 1 donne le récapitulatif des 06 cas pris en charge selon l'âge des patients, les circonstances de survenue, le délai de prise en charge, le motif de consultation, le diagnostic retenu et le type de traitement institué.

**Tableau 1:** Récapitulatif des différents cas de fractures de verge prise en charge

Patients	Age (ans)	Survenue	Délai de prise en charge	Motif de consultation	Diagnostic	Traitement
**N°1**	40	Faux pas du coït	5 heures	Tuméfaction verge+ douleur	Fracture de verge: lésion caverneuse	Albuginorraphie
**N°2**	43	Faux pas du coït	7 jours	Urétrorragie	Lésion urétrale + lésion spongieuse	Traitement conservateur: sonde urinaire
**N°3**	30	Manipulation forcée	10 jours	Tuméfaction verge+ douleur	Fracture de verge: lésion caverneuse	Albuginorraphie
**N°4**	35	Manipulation forcée	17 jours	Tuméfaction verge+ douleur	Fracture de verge: lésion caverneuse	Albuginorraphie
**N°5**	43	Faux pas du coït	3 heures	Urétrorragie+ tuméfaction de la verge +douleur	Fracture de verge: lésion caverneuse + contusion spongieuse	Albuginorraphie
**N°6**	39	Faux pas du coït	12 jours	Tuméfaction verge+ douleur	Fracture de verge: lésion caverneuse	Albuginorraphie

## Discussion

La fracture de verge est une urgence andrologique rare. Les différentes études comme celles de Natchagandé *et al.* [5] au Bénin et Kpatcha *et al.* [6] au Togo retrouvaient des séries proches de la nôtre, respectivement de 04 cas et de 06 cas sur 05 ans. Cependant, cette rareté dans la littérature n'est que le reflet d'une sous notification. Dans nos régions, il faut ajouter à cette sous notification, la méconnaissance de cette affection par les professionnels de la santé et la pudeur des patients empêchant certains de se faire consulter pour une telle lésion génitale [7]. En effet en 66 ans, Eke *et al.* [8] dans une revue de littérature dénombraient 1331 cas rapportés par 183 publications à travers le monde [6].

La moyenne d'âge de nos patients était de 38,3 ans avec des extrêmes de 30 et 43 ans. Elle était de 37,8 ans avec des extrêmes de 22 et 51 ans pour Barry MII *et al.* [7] et de 37,3 ans avec des extrêmes de 25 et 60 ans pour Kpatcha *et al.* [6]. Il s'agit d'une pathologie du sujet adulte à tout âge, en activité sexuelle, liée surtout à la vigueur et à la fréquence plus importante des rapports sexuels [8-10]. La cause la plus fréquente est le faux pas du coït lorsque le pénis en érection vient heurter le pubis lors des rapports sexuels entrainant une courbure exagérée de la verge [11]. Cette étiologie est la plus évoquée dans la littérature occidentale [12-14] ainsi que dans notre série. Cependant, dans certaines situations la manipulation forcée du pénis lors de manœuvre de masturbation ou toutes autres manœuvres forcées peut également être la cause de cet incident. Elle représentait 33% chez les patients dans la série de Ndiaye *et al.* [15] au Sénégal. Il s'agit de la situation observée dans deux cas de notre série. En effet Au moyen Orient, le faux pas du coït vient au second rang après la manipulation intempestive du pénis en érection lors des manœuvres masturbatoires, de retournement au lit pendant le sommeil ou de redressement et de camouflage d'une érection matinale dans un contexte de promiscuité [16-19].

Le délai de prise en charge est variable en général de quelques heures à quelques jours [7]. Il a été en moyenne de 46,3 heures avec des extrêmes de 03 et 408 heures, relativement long chez nos patients. De même, Kpatcha *et al.* [6] avaient trouvé un délai moyen relativement long de 74 heures qu'ils expliquaient par le fait qu'il existait une réticence des malades à consulter pour ce motif généralement difficile à évoquer en Afrique à cause de la pudeur.

Le diagnostic de fracture de verge était essentiellement clinique basé sur l'histoire stéréotypée de l'accident et l'examen physique caractérisé par une déformation de la verge « en aubergine » associée à une douleur pénienne. Il s'agit très souvent de la persistance de la douleur et surtout de la déformation monstrueuse de la verge qui amène le sujet à consulter. L'urétrorragie ou la rétention d'urine associée doit faire rechercher une rupture de l'urètre spongieux. Aucun examen para clinique n'est indispensable pour le diagnostic dans les formes typiques [8, 9, 14]. Cependant, dans les formes frustres ou vues tardivement certains auteurs recommandent l'échographie Doppler Couleur, l'urétrocystographie rétrograde (UCR), la cavernographie ou mieux l'imagerie par résonance magnétique (IRM) [9, 20-23], ceci fut le cas d'un de nos patients qui a bénéficié d'une échographie et d'un UCR. La rupture de l'albuginée et des corps caverneux est le plus souvent la règle. Il s'agit de la situation retrouvée dans 05 cas. La rupture isolée du corps spongieux est une entité rare. Elle peut être associée ou non à une rupture de l'urètre ou du corps caverneux [4]. Il s'agissait du tableau clinique décrit chez un de nos patients où on avait retrouvé une rupture du corps spongieux associée à une contusion de l'urètre. La place de l'échographie Doppler de la verge et de l'Imagerie par Résonance Magnétique (IRM) dans le faux pas du coït peut être nécessaire surtout en cas de rupture isolée des corps spongieux. L'échographie réalisée pourrait donner une caractérisation lésionnelle optimale, orienter le traitement et permettre de détecter d'éventuelles complications [2, 24, 25]. L'IRM est un examen très intéressant dans la recherche de la rupture des corps caverneux, sa sensibilité avoisinerait 100%, mais son utilisation est limitée par son coût .élevé [26].

La controverse entre traitement médicamenteux et chirurgical d'une fracture de verge n'est plus d'actualité. Bennani *et al.* [27] ont rapporté un taux de complications de 40,7% et 8,2% respectivement pour le traitement conservateur et celui chirurgical. La réparation chirurgicale en urgence est le meilleur moyen de prévention des complications. Selon Amer *et al.* [28], elle offrait moins de complications à type de dysfonction érectile, d'incurvation pénienne, de plaques ou de nodules que le traitement conservateur. Une autre approche consistant à différer la chirurgie entre le septième et le dixième jour a été préconisée par Nasser [29], l'auteur justifie cette attitude par le fait que ce délai permettrait la résorption de l''dème et de l'hématome ce qui rendrait la chirurgie plus aisée. L'abord coronal circonférentiel était notre préférence chez 05 de nos patients comme pour beaucoup d'autres auteurs [9, 16]. Cette voie d'abord permet une exploration exhaustive des lésions par rapport à la voie élective sur la zone de la fracture, mais qui a l'avantage d'être moins délabrant, réduisant ainsi le risque infectieux post opératoire. En Inde, Mahapatra *et al.* [30] ont rapporté deux cas de nécrose cutanée après déchaussement complet de la verge sur quinze patients traités par cet abord. Cinq de nos patients ont été traités par une incision circonférentielle dans le sillon balano-préputial et aucune complication infectieuse n'a été notée. Par contre un de nos patients a bénéficié d'un traitement conservateur du fait d'une rupture isolée de l'urètre spongieux avec un résultat satisfaisant. Selon certains auteurs [2, 7], devant une rupture isolée de l'urètre spongieux, le réalignement endoscopique doit être de mise.

Les séquelles de la fracture de verge sont représentées par la coudure de la verge en érection, la baisse de l'érection, la lésion nerveuse ou vasculaire et enfin la difficulté mictionnelle par rétrécissement urétral pouvant faire suite à une lésion urétrale. Dans notre série, bien que non exhaustive, aucun de nos patients n'avaient présenté de séquelles pour un suivi de six (06) mois. Par contre dans la série de Mahapatra *et al.* [30], sur 18 patients évalués après fracture de verge, 02 avaient une dysfonction érectile après un suivi de trois mois dont un présentait une insuffisance de l'artère caverneuse au Doppler et dans la série de Kpatcha *et al.* [6], deux patients avaient présenté des troubles érectiles marqués par le manque de rigidité de la verge et la difficulté de rester en érection jusqu'à la fin du rapport sexuel.

## Conclusion

La fracture de verge est une pathologie rare survenant le plus souvent chez l'adulte jeune au cours d'un rapport sexuel. Le diagnostic est pour la plus part du temps clinique. L'association d'une contusion du corps spongieux avec ou sans rupture urétrale doit être recherchée. Le traitement est dans la quasi-totalité des cas chirurgical et consiste à évacuer l'hématome sous-cutané et à suturer l'albuginée des corps caverneux.

### État des connaissances actuelles sur le sujet

- La fracture de verge est une pathologie rare et fait partie des urgences andrologiques;
- Le mode de survenue est là dans la majorité des cas le faux pas du coït entrainant la rupture des corps caverneux;
- La prise en charge en urgence consiste à la suture du corps caverneux rompu (albuginorraphie).

### Contribution de notre étude à la connaissance

- Notre série de cas montre la rareté de cette pathologie et surtout les points de similitude avec les données de la littérature;
- Nous avons noté un cas d'urétrorragie isolé due à une rupture isolée du corps spongieux entité très rare dans les fractures de verge;
- Cette série de cas permettra de faire le point de la prise en charge de cette pathologie au Burkina Faso, permettant ainsi de compléter la littérature dans le monde.

## Conflits d’intérêts

Les auteurs ne déclarent aucun conflit d'intérêts.
